# Call for candidates: Editor-in-Chief for *EJNMMI Reports*

**DOI:** 10.1186/s41824-025-00257-5

**Published:** 2025-07-12

The publisher, Springer Nature, is seeking applications and nominations for the position of the Editor-in-Chief (EiC) for the journal *EJNMMI Reports* (formerly known as European Journal of Hybrid Imaging).

*EJNMMI Reports* is the inclusive science journal of the EJNMMI journal family. It is a peer-reviewed open access journal. The editorial process of EJNMMI Reports does not focus on the extent of the advance of the submitted papers. All valid research with clinical and translational relevance in the field of Nuclear Medicine (including longer and brief reports, negative and positive results, provocative topics, data & protocol descriptors) will be considered, see formats considered below. Further details about the journal are available at https://ejhi.springeropen.com/.

*EJNMMI Reports* is a fully Open Access journal and considers scientific articles that are of interest to the nuclear medicine and molecular imaging community and cover diagnostics and therapy within all subfields in this subject area. The journal aims at providing clinical as well as scientific information that is useful for all relevant imaging and clinical communities as well as allied professions.

The journal is available in all major indexing services and has an impact factor. The journal publishes original research articles, brief communications on novel preliminary approaches, case reports, reviews, guidelines, editorials, images, and letters to the editor, published by experts from different clinical disciplines. Future article types will be fine-tuned by the EiC in consultation with the publisher.

The journal will be run under the leadership of one EiC who will be supported by Associate Editors, Section Editors, and an Editorial Board nominated by the EiC in close collaboration with the publisher. The EiC accounts for the content of the journal. This includes proactive content acquisition, as well as leading and managing the peer-review process in a timely manner in close cooperation with the Associate and Section Editors, the Editorial Board, Reviewers and Authors. In addition, the EiC should be interested in building strong relationships with experts in the field to proactively attract content and to arrange and commission special issues and/or collections on topics of interest to the international community of nuclear medicine and molecular imaging. The EiC adheres to the standards of good editorial practice, publication ethics and the publisher’s code of conduct.

The EiC serves for an initial term of three years. The term of service for this position starts on 1st January 2026 and expires on 31st December 2028.

The ideal candidate should have a vision on how to lead the journal, grow the published content and further increase the visibility and reputation of the journal within the international community. The EiC will establish a close working relationship with their Editors. The candidate should have a broad interest and strong expertise in nuclear medicine and molecular imaging as a whole, a high level of commitment to leading this journal and a clear idea of the pragmatic issues involved in accomplishing this mission. The EiC is expected to work closely with the global publisher Springer Nature on all aspects of publishing.

Each interested individual is invited to apply via email. The application should include a:

- brief letter of interest and qualifications.
- vision statement, including how they want to strategically develop the journal.
- short curriculum vitae, including publication list.
- full disclosure of conflicts of interest.

Contact for queries and applications: Sabine Ben Ghechir, Senior Publisher, Medicine and Life Sciences Journals at sabine.benghechir@springernature.com.

Submissions should be received by 15th September, 2025.

